# Factors Affecting Visual Prognosis of Myopic Foveoschisis after Macular Buckling

**DOI:** 10.1155/2022/9293347

**Published:** 2022-05-17

**Authors:** Xiujuan Zhao, Yanbing Wang, Yuqing Chen, Silvia Tanumiharjo, Yijin Wu, Ping Lian, Shida Chen, Xia Huang, Bingqian Liu, Lin Lu

**Affiliations:** State Key Laboratory of Ophthalmology, Zhongshan Ophthalmic Center, Sun Yat-sen University, Guangzhou 510060, China

## Abstract

**Purpose:**

To analyze the visual prognosis of macular buckling in patients with high myopia foveoschisis (FS) and to identify factors that predict the final visual outcome.

**Methods:**

We retrospectively included 155 eyes of 155 patients who underwent foveoschisis-related macular buckling. Best-corrected visual acuity (BCVA) and coexisting macular pathologies were assessed as a measure of surgical outcome, and multivariate linear regression was performed to identify factors affecting final visual prognosis.

**Results:**

The mean preoperative BCVA was 1.19 ± 0.55 logMAR (20/308), while the mean postoperative BCVA was 0.82 ± 0.51 logMAR (20/133) (*P* < 0.001). Anatomical success was achieved in 151/155 eyes (97.42%) after the first surgery and in 155/155 eyes (100%) at the 2-year follow-up visit. Both preoperative and postoperative BCVA were better in eyes without macular hole (MH) than in eyes with MH. In patients with MH, the postoperative BCVA was significantly better than that before surgery when the MH was closed. However, the difference was not significant in patients with unclosed MH. Univariate analysis identified that baseline BCVA, age, MH, atrophic myopic maculopathy category, and postoperative intraretinal cyst were significantly related to BCVA at the postoperative 2-year follow-up. Multivariate analysis revealed that preoperative BCVA and age were significant factors.

**Conclusion:**

Better preoperative BCVA and younger age are predictors of better prognosis. Prompt surgery is advised for patients with myopic foveoschisis to improve their visual prognosis.

## 1. Introduction

Myopic foveoschisis (MF) is a severe complication of pathological myopia that causes visual impairment. A combination of posterior scleral elongation and counteracting anterior vitreomacular traction of the retinal arterioles causes MF [1, 2]. Previous reports have shown encouraging results of pars plana vitrectomy (PPV) in resolving foveoschisis [3–7]. However, PPV cannot loosen the tractional forces induced by posterior staphyloma. Hence, it does not prevent the possible advancement of a macular hole (MH) [8], which is induced by posterior staphyloma while the vitreous cortex and retinal arterioles exert tangential traction [9–11]. Moreover, it is assumed that PPV induces further macular thinning by forcing the retina to adhere to the macular staphyloma, and the tangential traction and centrifugal traction generated postoperatively greatly increase the risk of the macular hole [12]. Macular buckling (MB) is required to release these tractional forces and has proven to be effective and safe in the treatment of MF in highly myopic eyes [12–14].

Advancing foveal detachment (FD) or MH formation can cause visual impairment in patients with MF. Hence, identifying factors affecting postsurgery visual outcomes is important to achieve favorable results. Some studies have identified prognostic factors significantly related to final visual results [15–17]. However, most of the studies published thus far have focused on the prognostic factors of PPV, while the data are lacking for MB surgery.

The aim of this study was to investigate the visual outcomes after MB surgery for MF and to identify prognostic factors related to BCVA at the postoperative 2-year follow-up.

## 2. Methods

This was a single-center investigation of 155 eyes from 155 patients who underwent MB for MF between January 2017 and February 2019. All surgeries were performed by a single surgeon (LL). MB was performed using a silicone sponge-titanium explant as had been previously described [14, 18]. This study was approved by the Ethics Committee of Zhongshan Ophthalmic Center and was conducted in accordance with the principles of the Declaration of Helsinki and applicable local regulations. This study was registered on the website ClinicalTrials.gov with ID NCT03433547. Informed consent was obtained from all patients. The following inclusion criteria were adopted: (1) patients with the diagnosis of MF; (2) patients who had undergone MB for MF treatment; and (3) a refractive error with a spherical equivalent (SE) ≤ -6.0 diopters and/or axial length (AL) ≥ 26.5 mm. The exclusion criteria were as follows: (1) patients with any intraocular surgery history; (2) patients with dense cataract, advanced glaucoma, or amblyopia; (3) patients with a history of myopic choroidal neovascularization (mCNV); and (4) macular detachment extending to the peripheral retina. Only data corresponding to the surgical eye of each patient were used for the statistical analyses.

**Figure 1:**
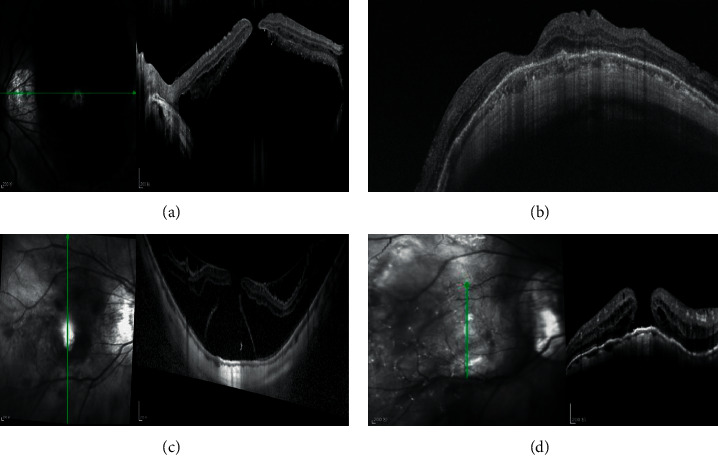
Presurgery and postsurgery OCT images of patients with MH and MD. (a) Presurgery image of MH and MD. (b) Image of MH closure at 23-month follow-up postsurgery. (c) Presurgery image showing MH, foveoschisis, and MD. (d) Image showing retinal reattachment but not MH closure at the 25-month follow-up postsurgery. MD = macular detachment, MH = macular hole, and OCT = optical coherence tomography.

All patients underwent the following examinations: (1) BCVA assessment with refraction; (2) measurement of AL with the Intra Ocular Lens Master (Carl Zeiss, Tubingen, Germany); (3) fundus photography (fundus camera TRC-50; Topcon, Tokyo, Japan); and (4) spectral-domain optical coherence tomography (SD-OCT) of the macula (Heidelberg Engineering, Heidelberg, Germany; DRI-OCT, Topcon Corp, Tokyo, Japan) before and after the surgery. Examination results obtained at 6 months, 1 year, and 2 years postoperatively were analyzed. Anatomical success was defined as the complete reattachment of the foveoschisis and any related FD. MH closure was defined as the recovery of normal anatomy without any remaining detachment of the inner retinal layers at the foveal region found on any optical coherence tomography (OCT) scan (Figure 1). The ATN classification and grading system defines atrophic myopic maculopathy (AMM) using the following categories [19]: A1, tessellated fundus only; A2, diffuse chorioretinal atrophy; A3, patchy chorioretinal atrophy; and A4, complete macular atrophy. Myopic traction maculopathy (MTM) is classified as follows: T0, no macular schisis; T1, inner or outer foveoschisis; T2, inner and outer foveoschisis; T3, foveal detachment; T4, full-thickness MH; and T5, MH with retinal detachment.

Statistical analysis was performed using SPSS statistical software (version 22.0; IBM Corp Released 2013. IBM SPSS Statistics for Mac, IBM Corp., Armonk, NY, USA). All values are expressed as mean ± SD or proportions as appropriate. The VA was compared between the two groups using independent sample *t*-tests. Univariate and multivariate linear regression analyses were both performed to evaluate the possible predictive factors associated with postoperative BCVA. Statistical significance was indicated by a *P* value of less than 0.05.

## 3. Results

### 3.1. Baseline Characteristics

A total of 173 consecutive highly myopic patients (173 eyes) were initially selected for the study; however, 18 eyes were excluded due to missing data. A total of 155 eyes from 155 patients were finally included in the study, and their demographics and clinical characteristics are shown in Table 1. Out of 155 eyes, 52 eyes showed foveoschisis with an MH and 103 eyes showed foveoschisis without an MH. The severity of AMM was classified as A1 in 7 eyes, A2 in 115 eyes, A3 in 27 eyes, and A4 in 6 eyes. The MTM included 5 eyes with T2, 98 eyes with T3, 10 eyes with T4, and 42 eyes with T5.

### 3.2. Overall Anatomical and Functional Changes

Anatomical success was attained in 151/155 eyes (97.42%) after the first surgery and in 155/155 eyes (100%) at the 2-year follow-up, with 2 patients underwent a second PPV surgery due to retinal detachment, while the buckle of two patients had to be readjusted due to improper positioning. In those without an MH, initial success was achieved with one-time surgery in 102/103 eyes (99.03%) and in 103/103 eyes (100%) at the 2-year follow-up. In eyes with an MH, success after the first surgery was achieved in 49/52 eyes (94.23%), and the MH closure rate was 71.15% (37/52 eyes).

The BCVA at the 2-year follow-up was significantly better than that at presurgery BCVA, regardless of the presence or absence of MH (*P* < 0.01). Both the pre- and postsurgery BCVA results were better in eyes without MH than in eyes with MH (*P* < 0.05). Although the improvement in BCVA was not significantly different between the two groups (*P*=0.762), the BCVA at the 2-year follow-up was significantly better in eyes without MH than in eyes with MH (*P*=0.004) (Table 2).

A significant improvement from preoperative to postoperative BCVA was noted in eyes with MH closure (*P*=0.003), but not in eyes without MH closure (*P*=0.091). However, neither the change from pre- to postsurgery BCVA nor the BCVA improvement between MH closure and nonclosure were significant (*P* > 0.05) (Table 3).

There were 74 eyes with preoperative BCVA better than or equal to logMAR 1.0 and 81 eyes with preoperative BCVA worse than logMAR 1.0. The presurgery BCVA values in eyes with better preoperative VA were 0.69 ± 0.23, whereas those in eyes with worse preoperative VA were 1.64 ± 0.35 (*P* < 0.001). Those eyes with BCVA ≥ logMAR 1.0 at baseline showed a statistically significant difference between pre- and postoperative BCVA (1.64 ± 0.35 vs. 1.11 ± 0.56, *P* < 0.001). However, those eyes with BCVA < logMAR 1.0 at baseline showed no statistically significant difference between pre- and postoperative BCVA (0.69 ± 0.23 vs. 0.66 ± 0.45, *P*=0.601). Additionally, we found that the group with better baseline BCVA indeed obtained a better postoperative BCVA with a statistical significance (0.66 ± 0.45 vs. 1.11 ± 0.56, *P* < 0.001) (Table 4).

### 3.3. Predicting Factors for Postoperative BCVA

**Figure 2:**
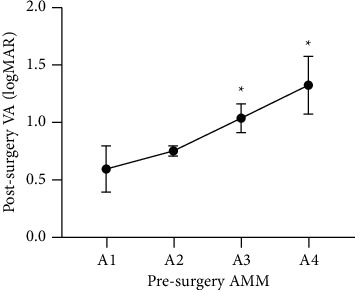
The relationship of presurgery AMM and postsurgery VA (logMAR). It showed a notable increasing trend from A1 to A4 (*P* = 0.002, one-way analysis of variance) and significant differences between A3 or A4 and A1 or A2 (P_A1-A3_ = 0.034, P_A2-A3_ = 0.007, P_A1-A4_ = 0.009, and P_A2-A4_ = 0.006, Fisher's least significant difference) (^*∗*^). AMM = atrophic myopic maculopathy and VA = visual acuity.

**Figure 3:**
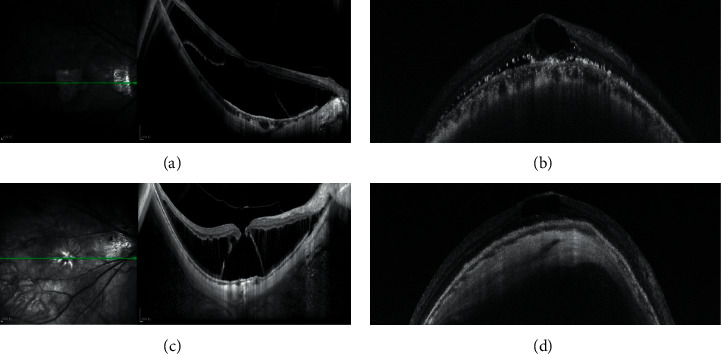
Presurgery and postsurgery OCT images of patients with postoperative intraretinal cysts. (a) Presurgery image showing foveoschisis with MD. (b) Image of intraretinal cyst at 21 months postsurgery. (c) Presurgery image of MH with MD. (d) Image of intraretinal cyst at 24 months postsurgery. MD = macular detachment, MH = macular hole, and OCT = optical coherence tomography.

To identify factors potentially affecting the prognosis of BCVA at the 2-year follow-up, linear regression analyses were performed. Univariate linear regression analysis showed that age, preoperative BCVA, MH, AMM category (Figure 2), and presence of intraretinal cyst (Figure 3) were statistically significant parameters in relation to postsurgery visual function (*P* < 0.001, *P* < 0.001, *P*=0.004, *P* < 0.001, and *P*=0.039, respectively). However, there was no change in the AMM category of each patient during the two years of observation after the surgery. To evaluate the association between these significant parameters and postoperative BCVA, a multivariate linear regression analysis was performed. Only age and preoperative BCVA remained significantly related to postoperative BCVA (*P*=0.002 and *P* < 0.001, respectively) (Table 5).

### 3.4. Complications

After macular buckling, patients tended to have a variable elevation of intraocular pressure within the first month, but all recovered to normal levels within three months using pressure-lowering agents or glucocorticoids. Three patients with a postoperative vitreous hemorrhage and five eyes with epiretinal hemorrhage were observed although all the hemorrhages were self-limiting and completely absorbed within three months without any treatment. No postoperative infections occurred. Almost all the patients exhibited some degree of surgically induced eye movement disorder, strabismus, metamorphopsia, and binocular diplopia. However, the symptoms were gradually reduced or fully resolved on their own without medication within two years. Postoperative intraretinal cyst was seen in 17 eyes, but no improvement was observed over time.

## 4. Discussion

In this study, we aimed to identify factors that predict the prognosis of visual function after MB in highly myopic foveoschisis. Better preoperative BCVA and younger age were significantly correlated with better visual outcomes.

MB is an effective and safe intervention for the treatment of MF in highly myopic eyes [13, 14, 18]. Unlike PPV, MB can counter the outward expansion of the eyeball and address the global cause of traction. Previous studies [13, 14] showed that MB achieved higher initial anatomic success and better functional outcomes than PPV in highly myopic MF and MH. Parolini suggested that MB should be applied as the first approach in myopic traction maculopathy while PPV could be served as a second surgery to treat the epiretinal membranes and the foveal abnormalities [12].

Our study revealed that better preoperative BCVA and younger age are indicative of good postsurgery outcomes in visual function. Several studies have identified prognostic factors for PPV in highly myopic foveoschisis [16, 20, 21]. Lim et al. [16] found that presurgical ellipsoid zone (EZ) disruption and thinner central foveal thickness in MF patients are linked to poorer prognosis after PPV. EZ was not a factor of analysis in our study, as most OCT scans did not allow assessing EZ disruption due to the extreme AL in highly myopic eyes. An investigation by Lehmann et al [21] found that preoperative visual acuity was the most influential factor for final visual acuity, while Fujimoto et al. [20] showed that changes in retinal thickness and the recovery of photoreceptor cells seen in OCT scans were significantly related to final postsurgery BCVA. Hence, some possible prognostic factors mentioned in other studies were also analyzed in our study. We found that better baseline BCVA and younger age were pivotal in predicting visual prognosis. Better preoperative BCVA is indicative of greater preservation of retinal neuronal function; hence, achieving better visual recovery is more likely after surgery.

There was no independent correlation between final BCVA and other presurgical factors, including AL, refractive error, and FD. Previous studies [22, 23] have indicated that a shorter AL is related to better visual recovery and higher initial reattachment rate after PPV surgery. However, the results of our study showed that the outcome of visual recovery was not related to AL. A possible explanation is selection bias, which might have occurred to some extent in our series, as most of our patients had a much longer AL compared with patients in other studies. Furthermore, it can be postulated that MB surgery counteracted the posterior sclera expansion, thereby reducing the extent of relative lack of the retina. The existence of FD was found to be correlated with greater visual improvement or better final BCVA in some studies [22, 24]. However, Kim et al. [25] showed that presurgical FD is often correlated with poor functional and anatomical outcomes. Our analysis did not find a correlation between FD and final BCVA, possibly because most patients (90.32%) in our study had presurgical FD. On the other hand, the AMM category also appeared to be correlated with final BCVA. Continuous extension of the posterior region affected the extent of myopic degeneration; hence, destruction of the inner and outer retinal structures due to progressive distention could cause more permanent damage that would impede visual recovery. In all postsurgical patients, AMM category has not progressed over 2 years of observation. Given that macular buckling could restore the anatomical position of the retina and improve choroidal and retinal blood circulation, nutrition and oxygen supplementation from the circulation seemed to compensate for retinal repair and halt the progression of atrophic lesions [26]. Lehmann et al. [21] suggested that a reduced BCVA of 20/40 Snellen equivalent in myopic foveoschisis was an indicator of surgery requirement. However, we cannot arrive at a similar conclusion in this study, as presurgery BCVA was much lower in our series (1.19 ± 0.55 vs. 0.68 ± 0.37). Our study concluded that eyes without MH could maintain a better final BCVA than eyes with MH. After MH formation, there was no significant difference in final BCVA regardless of whether the MH was closed after surgery. This suggests that prompt surgical treatment is pivotal in gaining better visual recovery in foveoschisis patients, especially if performed before the formation of MH.

In our study, postoperative intraretinal cyst was seen in 17 eyes (10.97%) but in none after PPV surgery [13]. 47.06% (8/17) of these cases had presurgical MH. The presence of cysts was seen as cystoid changes in the outer nuclear layer (ONL) without subretinal fluid. Low vision was associated with severe thinning of the retina at the central macula, indicating loss of retinal tissue and massive death of foveal cells. An intraretinal cyst masked a severe retinal macular degeneration, as the retinal thickness appeared to be normal in these cases. Retinal degeneration was thought to be accompanied by ONL thinning. Poor retinal pigment epithelium function due to extremely high myopia might be the cause of retinal degeneration and failure of retinal outflow mechanisms, explaining the presence of postsurgery intraretinal cysts. The surgery-related complications included transient elevation of intraocular pressure, eye movement disorder, strabismus, metamorphopsia, and binocular diplopia. They were usually related to edema and injury to soft tissues such as muscles, as well as tension and height of the inward bulge from the buckle, which largely resolved as the postoperative inflammation subsided and the height of the buckle gradually decreased [18]. Meanwhile, it was possible that subretinal fluid was gradually absorbed as the restoration of the microcirculatory drainage due to the reduced compression force of the buckle [27].

There were some limitations in our study. First, this was a retrospective study. Second, selection bias and allocation bias cannot be discarded, and the surgeon's preferences, experience, and skills might have affected the choices made during the surgical procedure and therefore the outcome. Third, variability would be expected at the level of indentation for each patient in terms of relative height and shape, as these performances were under the subjective judgement of the surgeon through indirect ophthalmoscopy. Fourth, two different types of OCT were used in our study, which might have an influence on the judgement due to the difference in their procedures though they have the same diagnostic capability.

In conclusion, the main factors affecting postsurgery BCVA were preoperative BCVA and age. Current maneuvers in MB surgery are highly effective for foveal reattachment.

## Figures and Tables

**Table 1:** Demographics and characteristics of the patients with myopic foveoschisis at baseline visit.

Parameter	Value
Number	155
Age (years)	56.79 ± 11.48
Sex (male/female)	45/110
Refractive error (D)	−12.96 ± 4.77
Preoperative AL	29.74 ± 2.00
Preoperative logMAR VA (Snellen)	1.19 ± 0.55
Postoperative refractive error	−5.40 ± 6.84
Postoperative AL	26.66 ± 2.38
Postoperative logMAR VA (Snellen)	0.82 ± 0.51
With macular hole	52
Without macular hole	103
AMM (A1/A2/A3/A4)	7 (4.52%)/115 (74.19%)/27 (17.42%)/6 (3.87%)
MTM (T2/T3/T4/T5)	5 (3.23%)/98 (63.23%)/10 (6.45%)/42 (27.10%)

D: diopters, AL: axial length, VA: visual acuity, AMM: atrophic myopic maculopathy, and MTM: myopic traction maculopathy.

**Table 2:** Comparison of BCVA in eyes with or without MH.

	MF with MH	MF without MH	*P*
Preoperative BCVA	1.34 ± 0.56	1.12 ± 0.53	0.018
Postoperative BCVA	0.98 ± 0.46	0.73 ± 0.51	0.004
BCVA gain	−0.36 ± 0.55	−0.38 ± 0.47	0.762
*P*	0.001	<0.001	

BCVA: best-corrected visual acuity, MH: macular hole, and MF: myopic foveoschisis.

**Table 3:** Comparison of BCVA in eyes with or without MH closure.

	With MH closure (37)	Without MH closure (15)	*P*
Preoperative BCVA	1.37 ± 0.57	1.27 ± 0.56	0.596
Postoperative BCVA	0.99 ± 0.48	0.96 ± 0.42	0.791
BCVA gain	−0.38 ± 0.59	−0.31 ± 0.44	0.637
*P*	0.003	0.091	

BCVA: best-corrected visual acuity and MH: macular hole.

**Table 4:** Preoperative and postoperative BCVA of two groups separated by baseline visual acuity.

	BCVA < logMAR 1.0	BCVA ≥ logMAR 1.0	*P*
Number	74	81	
Preoperative BCVA	0.69 ± 0.23	1.64 ± 0.35	<0.001
Postoperative BCVA	0.66 ± 0.45	1.11 ± 0.56	<0.001
*P*	0.601	<0.001	

BCVA: best-corrected visual acuity.

**Table 5:** Multiple linear regression analysis of preoperative and postoperative factors with visual acuity at 2 y after surgery.

	Univariate		Multivariate	
	*β* (95% CI)	*P*	*β* (95% CI)	*P*
Age	0.374 (0.01, 0.023)	<0.001	0.219 (0.004, 0.016)	0.002
Preoperative BCVA	0.561 (0.396, 0.640)	<0.001	0.450 (0.291, 0.540)	<0.001
Preoperative RE	0.036 (−0.013, 0.021)	0.657		
Preoperative AL	0.105 (−0.014, 0.067)	0.194		
*Preoperative findings*				
MH	0.231 (0.081, 0.416)	0.004	0.104 (−0.030.0.253)	0.121
AMM category	0.279 (0.111, 0.381)	<0.001	0.103 (−0.027, 0.209)	0.128
Foveal detachment	−0.077 (−0.406, 0.141)	0.341		
*Postoperative OCT factors*				
Intraretinal cyst	−0.166 (−0.447, −0.011)	0.039	−0.095 (−0.307, 0.044)	0.140
Subretinal fluid	−0.034 (−0.343, 0.223)	0.678		
MH closure	−0.158 (−0.444, 0.124)	0.263		

BCVA: best-corrected visual acuity, RE: refractive error, AL: axial length, MH: macular hole, AMM: atrophic myopic maculopathy, and OCT: optical coherence tomography.

## Data Availability

The data that support the findings of this study are available from the corresponding author upon reasonable request.
